# Surgical repair of torn base of ruptured middle cerebral artery trifurcation aneurysm

**DOI:** 10.1007/s00701-024-06016-y

**Published:** 2024-03-25

**Authors:** Rui Zhang, Sajjad Muhammad

**Affiliations:** 1 Department of Neurosurgery, Medical Facultyand , University Hospital Düsseldorf, Heinrich-Heine-University, Moorenstrasse 5, 40225 Dusseldorf, Germany; 2 Department of Neurosurgery, University of Helsinki and Helsinki University Hospital, Helsinki, Finland; 3 Department of Neurosurgery, Xingtai People’s Hospital Hebei Medical University, Xingtai, China; 4 Department of Neurosurgery, King Edward Medical University, Lahore, Pakistan

**Keywords:** Microsurgical suturing technique, Intracranial aneurysm clip reconstruction, Revascularization, Subarachnoid hemorrhage

## Abstract

**Background:**

Treating complex middle cerebral artery (MCA) trifurcation aneurysms requires a delicate balance between achieving aneurysm obliteration and preserving vascular integrity. Various cerebral revascularization techniques, including bypass, and clip reconstruction are considered individually or in combination.

**Methods:**

This case report outlines a successful repair of a ruptured neck and base of MCA trifurcation aneurysm using a suturing-clip reconstruction technique. Temporary aneurysm trapping was implemented, with maintained elevated blood pressure to ensure collateral perfusion during repair of ruptured base and neck of MCA aneurysm.

**Conclusion:**

The suturing-clip reconstruction exhibited long-term radiological stability, emerging as a valuable alternative for managing challenging MCA trifurcation aneurysms.

**Supplementary Information:**

The online version contains supplementary material available at 10.1007/s00701-024-06016-y.

## Relevant surgical anatomy

The MCA, a vital component of the cerebral vasculature, comprises distinct segments including M1, M2, M3, and M4. The conversion of M1 into several branches (M2) creates intricate hemodynamic conditions, potentially contributing to aneurysm formation [5]. The dynamic blood flow and shear forces exerted on the arterial wall in this region may precipitate the development of aneurysms, particularly at the M1-M2 junction [2]. Notably, the vicinity of MCA aneurysms often involves perforators originating from the aneurysm base or near the aneurysm neck on the parent artery. This anatomical complexity, for example, a trifurcation of MCA, adds a layer of intricacy to surgical clipping [3]. In the event of an aneurysm rupture, subarachnoid hemorrhage (SAH) and hematoma formation with a swollen brain further amplify the surgical challenge. Surgical management options, including surgical clipping, endovascular coiling, clip reconstruction, or aneurysm trapping with bypass procedures, aim to isolate the aneurysm from normal circulation. The choice between these interventions is often made after careful consideration of the specific anatomical and clinical features of the MCA aneurysm [4, 6, 7].

## Description of the technique

### Preoperative assessment

**Fig. 1:**
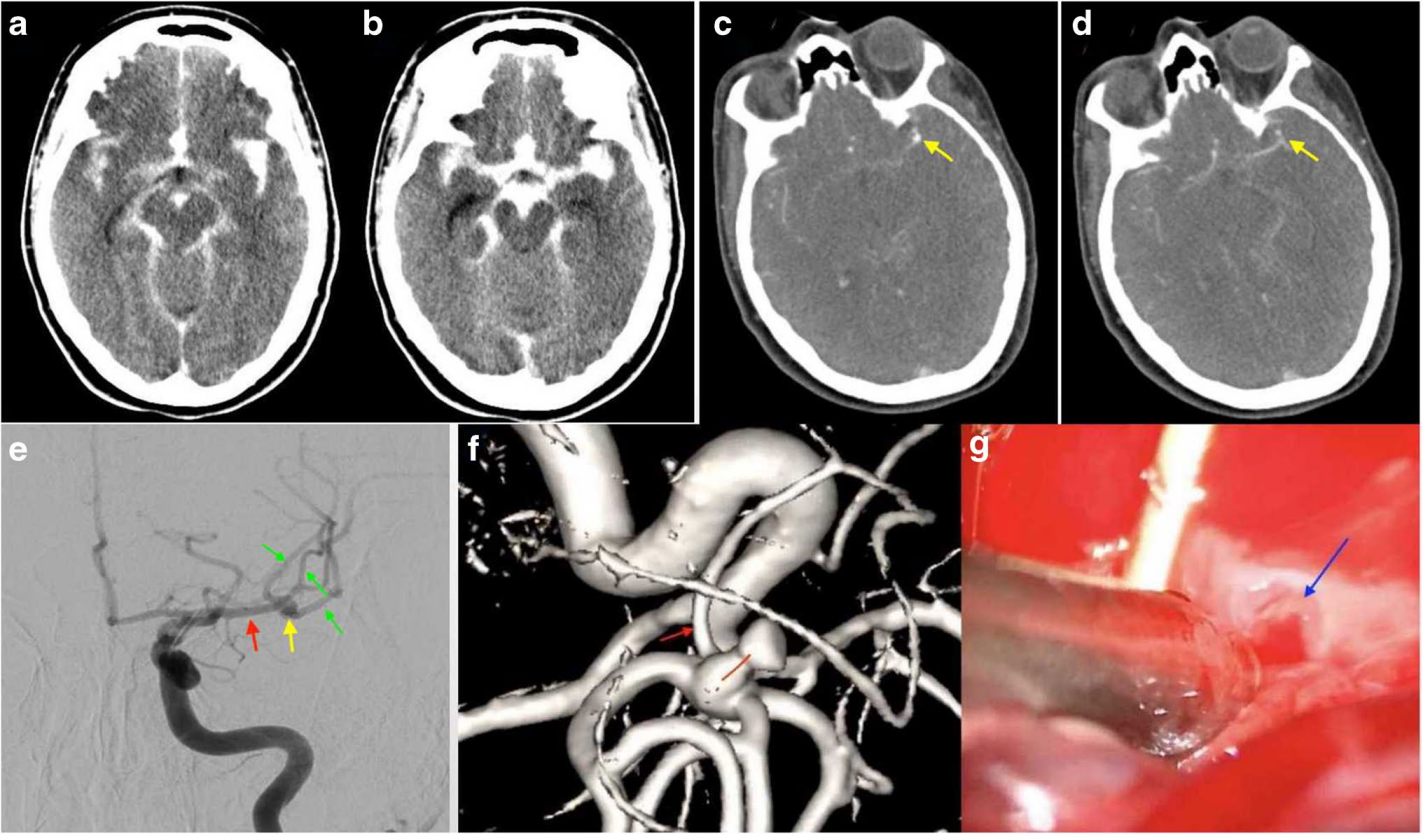
Preoperative imaging assessment of the MCA aneurysm. **A** and **B** A preoperative CT scan reveals a substantial subarachnoid hemorrhage, accompanied by the presence of a cisternal blood clot. **C** and **D** Preoperative CTA scan providing insight into the suspected position of the MCA aneurysm. **E** Preoperative DSA confirms the precise location of the aneurysm (indicated by the yellow arrow), with additional annotations highlighting M1 (red arrow) and M2 (green arrow). **F** Visualization of the partially torn neck of the aneurysm (depicted by the red line). Noteworthy is the proximity of a perforator artery originating from the M2 branch, emphasized by the red arrow. **G** Detailed view of the torn site of the aneurysm base, with the delicate and vulnerable area highlighted by the blue arrow

A 49-year-old female patient presented with a sudden and severe headache, and upon admission, she exhibited a GCS of 11 (WFNS Grade IV). Immediate imaging diagnostics, including Computed Tomography (CT) and CT angiography (CTA), revealed a Fischer grade III SAH. Multiple aneurysms were evident on digital subtraction angiography (DSA). The left MCA trifurcation aneurysm was identified as the probable source of the SAH. Due to a seizure and reduced GCS of 8, the patient was intubated and external ventricular drainage (EVD) was placed immediately. Given the complexity of the MCA trifurcation aneurysm, an interdisciplinary decision favored surgical repair (Fig. 1).

### Surgical procedure

The patient was positioned supine with the head rotated 60° to the right, elevated above heart level, and secured with a Mayfield holder. A left-sided pterional skin incision and standard pterional craniotomy were performed [9]. Dura was opened in curvilinear fashion. Microscopic dissection along the sphenoid ridge and opening of the optic cistern provided access.

For proximal control, dissection along the internal carotid artery (ICA) revealed the terminal of the ICA and the M1 branch of the MCA. The Sylvian fissure was widely opened to expose the M1 trunk and all M2 branches. Intraoperative, an aneurysm re-rupture occurred. Proximal and distal clipping of all M2 branches isolated the aneurysm, and blood pressure was elevated to improve collateral circulation.

Due to the extent of the ruptured area over the trifurcation base, direct clipping without compromising the parent artery was unfeasible. Consideration was given to M2-M2 side-to-side anastomosis and clip reconstruction. To mitigate the risk of ischemia in case of bypass failure, an interrupted suturing of the ruptured base was performed using 10–0 Nylon sutures. The aneurysm was then clip-reconstructed.

**Fig. 2:**
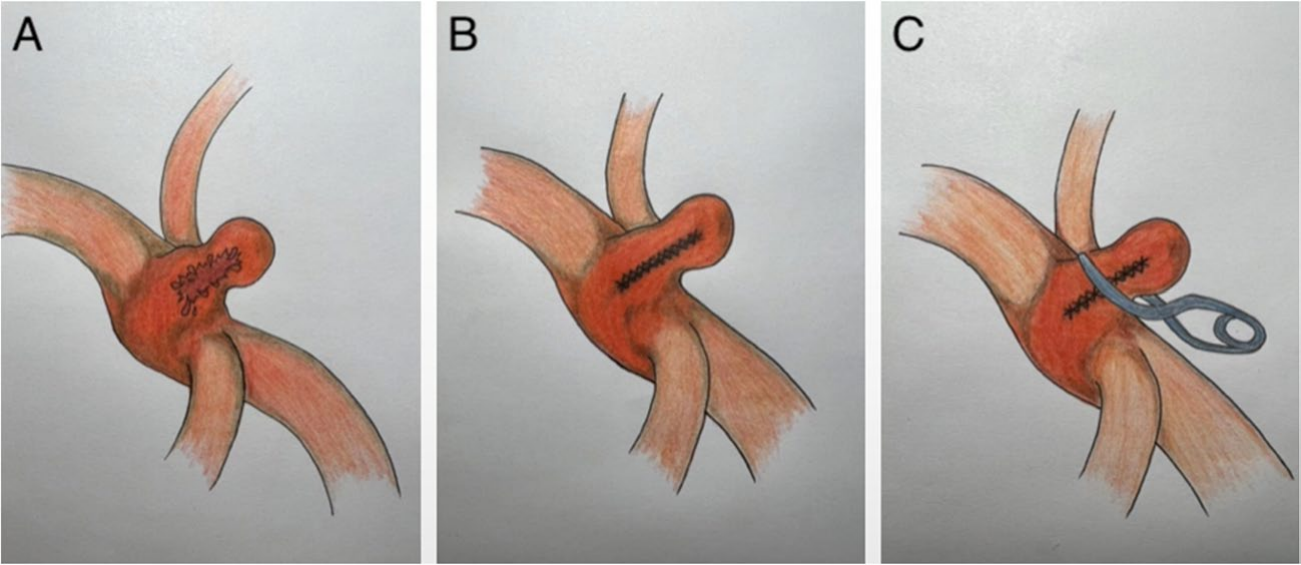
Schematic presentation of the suturing/reconstruction technique. **A** Presentation of rupture line over the aneurysm base and the parent artery wall junction. **B** Reconstruction of the fragile base of the aneurysm with interrupted suturing and obliteration of the vulnerable vascular area. **C** Showing the clip application to reconstruct the sutured base keeping the patency of the parent vessel

Upon releasing the distal clips, the suture line extending into the trifurcation was monitored. Subsequently, the proximal clip was released, and the patency of M2 vessels and stability of the sutured trifurcation area was ensured and no leakage occurred from the suture site. Doppler examination and indocyanine green (ICG) angiography confirmed optimal blood flow in all M2 branches and the absence of aneurysm perfusion. The sutured site was further secured with Tachosil, a muscle patch, and fibrin glue. Standard closure procedures for the dura, muscle, and scalp were carried out. This comprehensive approach ensured the successful repair of the ruptured MCA trifurcation aneurysm while minimizing the risk of ischemia and achieving excellent vascular and aneurysm perfusion outcomes (video. 1; Fig. 2).

### Indications

This report details a successful repair of the ruptured base of an MCA trifurcation aneurysm using a combined suture and clipping technique. The success of the procedure hinges on several key factors, including the early establishment of proximal control, careful maneuvering to ensure a wide opening of the Sylvian fissure while protecting perforators, and achieving thorough aneurysm obliteration while preserving the parent vessel. This approach serves as a viable option, particularly in scenarios with time constraints and the potential for extended ischemia. Moreover, this technique can be applied if the bypass suturing expertise are lacking to rescue such complex situations.

## Limitations

1. Surgeon expertise

### Skill requirement

The technique demands a high level of surgical skill, particularly deep suturing. Novice surgeons may face challenges in mastering this intricate skill, potentially affecting the procedure’s success.

### Anatomical familiarity

In-depth knowledge of complex anatomical structures is essential. Lack of experience may lead to encounter difficulties navigating the intricate vascular anatomy, increasing the risk of complications.

### Emotional preparedness

Strong emotional resilience is imperative for managing emergencies during the procedure, emotional preparedness particularly to remain composed in unexpected critical scenarios, potentially impacting decision-making and patient outcomes.

2. Equipment Demands

### Microscope requirements

The technique relies on a high-resolution microscope, necessitating excellent optics and features like an intraoperative assessment with ICG functionality. Facilities lacking such advanced microscopes may face limitations in implementing the technique effectively.

### Microsurgical instruments

The use of specialized microsurgical instruments is essential to perform deep suturing. Availability and accessibility to these instruments may pose a challenge in some settings, restricting the widespread application of the technique.

3. Team cooperation

### Anesthesia and nursing support

Successful execution of the technique relies on a well-coordinated and cooperative team. Swift responses from anesthetists and nurses are crucial in managing unforeseen scenarios. In environments where teamwork is not optimal, the technique’s feasibility may be compromised, potentially impacting patient safety.

## How to avoid complications

**Fig. 3:**
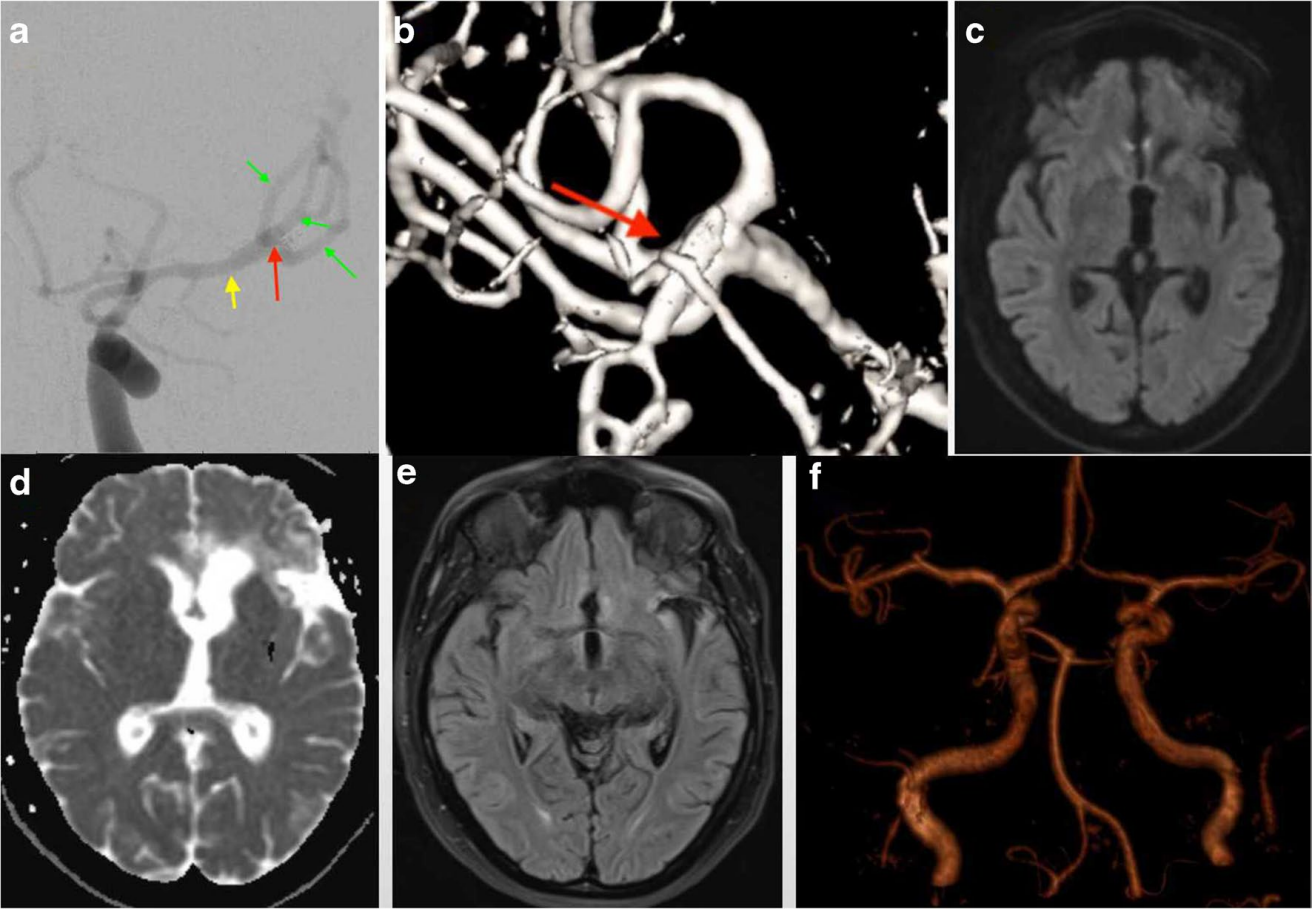
Postoperative assessment and long-term follow-up. **A** Postoperative DSA illustrating successful aneurysm elimination and confirming the patency of both parent and distal arteries. The position of the clip is denoted by the red arrow, while M1 and M2 branches are indicated by the yellow and green arrows, respectively. **B** Postoperative 3-dimensional angiography offers a comprehensive view, highlighting the complete elimination of the aneurysm (red arrow) and ensuring normal patency of all MCA branches. **C**, **D**, and **F** Two-year follow-up magnetic resonance (MR) images showcasing the absence of significant discernible deficits or abnormalities in the brain parenchyma. Modalities include T1 scan (C), diffusion-weighted imaging (DWI) (E), and fluid-attenuated inversion recovery (FLAIR) (F). **G** Long-term follow-up magnetic resonance angiography (MRA) image captured after 2 years, revealing sustained elimination of the aneurysm and demonstrating a stable course of the sutured/reconstructed trifurcation area. No evidence of recurrent aneurysm formation is observed

To mitigate potential complications, our surgical approach involves a strategic combination of techniques. Temporary clipping, maintained for approximately 25 min along with induced hypertension to 160 mmHg, is employed to safeguard against ischemia, preserving collateral circulation during the procedure [8]. Intraoperative measures include meticulous observation of the anastomosis site and real-time confirmation using ICG to ensure the obliteration of aneurysm perfusion, reducing the risk of leakage. Furthermore, a vigilant long-term follow-up plan is implemented to monitor the vulnerable artery site, minimizing the potential for aneurysm recurrence and enabling timely intervention if required. These multifaceted strategies collectively aim to enhance the safety and success of the surgical procedure while addressing the key complications of ischemia and postoperative bleeding or aneurysm recurrence (Fig. 3).

## Specific information for the patients

For patients considering this technique, it is vital to understand that while it effectively eliminates the aneurysm, it does not address the preexisting SAH. The ultimate outcome is significantly influenced by the severity of the SAH. Regular health check-ups, timely detection of intracranial aneurysms, and proactive monitoring to predict the risk of rupture are crucial for overall management [1]. Additionally, patients should be aware that the suture site on the artery wall is a vulnerable area with short-term leakage and long-term recurrence risks. Perioperative monitoring and diligent long-term follow-up are indispensable to ensure early detection and prompt intervention if needed, contributing to the success and safety of the procedure over time.

## Supplementary Information

Below is the link to the electronic supplementary material.

Supplementary file1 (MOV 286484 KB)

## Data Availability

All data and materials used in this study are available upon reasonable request. Please contact Sajjad Muhammad at sajjad.muhammad@med.uni-duesseldorf.de for any inquiries regarding the availability of data and materials.

## Abbreviations

MCA: Middle cerebral artery
SAH: Subarachnoid hemorrhage
GCS: Glasgow Coma Scale
WFNS: World Federation of Neurological Surgeons
CT: Computed tomography
CTA: Computed tomography angiography
DSA: Digital subtraction angiography
EVD: Extra ventricular drainage
ICA: Internal carotid artery
ICG: Indocyanine green
MR: Magnetic resonance
DWI: Diffusion-weighted Imaging
FLAIR: Fluid-attenuated inversion recovery
MRA: Magnetic resonance angiography
SSEP: Somatosensory-evoked potentials
MEP: Motor-evoked potentials
